# Endometriosis and dysbiosis: State of art

**DOI:** 10.3389/fendo.2023.1140774

**Published:** 2023-02-20

**Authors:** Brunella Zizolfi, Virginia Foreste, Alessandra Gallo, Simona Martone, Peirluigi Giampaolino, Attilio Di Spiezio Sardo

**Affiliations:** ^1^ Department of Public Health, University of Naples Federico II, Naples, Italy; ^2^ Department of Molecular and Developmental Medicine, University of Siena, Siena, Italy

**Keywords:** endometriosis, dysbiosis, microbiota, estrobolome, infertility

## Abstract

Endometriosis is a complex and heterogeneous disease affecting approximately 10% of reproductive age women. The hypothesis that alterations in the microbiota are involved in the pathogenesis of endometriosis has been postulated. Possible explanations for the implications of dysbiosis in endometriosis include the Bacterial Contamination hypothesis and immune activation, cytokine-impaired gut function, altered estrogen metabolism and signaling. Thus, dysbiosis, disrupt normal immune function, leading to the elevation of proinflammatory cytokines, compromised immunosurveillance and altered immune cell profiles, all of which may contribute to the pathogenesis of endometriosis. The aim of this review is to summarize the available literature data about the relationship between microbiota and endometriosis.

## Introduction

Endometriosis is a chronical gynecological disease characterized by the presence of ectopic endometrium growing outside the uterine cavity (1). It is thought to affect 10-15% of women of reproductive age, and its incidence rises among infertile women (2). Ectopic endometrium is mostly found in the ovaries, but it can spread into pelvic peritoneum, uterosacral ligaments, fallopian tubes and broad ligaments (3). Although more unusual, it can be also found in abnormal locations both within and outside the pelvis. Women who suffer from endometriosis, typically, experiment dysmenorrhea, chronic pelvic pain, dyspareunia together with other complication, among which infertility. Endometriosis is found in approximately 25 to 50% of infertile women, and 30 to 50% of endometriosis patients have difficulties to become pregnant (2). Different mechanisms are potentially involved in infertility impairment, including anatomical and microenvironmental conditions that may negatively impact the oocyte competence acquisition, egg fertilization, zygote transport within the tube and embryo implantation. It is therefore a condition that severely affects the quality of life, impacting on physical as well as mental, sexual and social wellbeing.

The ectopic implants respond to hormonal estrogenic stimulation and are driven to proliferate and bleed during the menstrual cycle (4, 5), due to high levels of estrogen receptor- *β* (HER- *β*); and endometriosis has been associated with alteration in estrogen signaling (6, 7). The cyclical bleeding activates local inflammation, inducing, at long term, fibrosis and chronical pelvic pain. The pathogenesis of endometriosis is still unclear. The most accepted theory is Sampson’s retrograde menstruation, according to which, menstrual blood flows through the salpinges into the peritoneal cavity (8).

In 1987, Gleicher et al. presented the theory that endometriosis may be an autoimmune disease considering that most of the criteria of autoimmune diseases, including polyclonal B cell activation, immunological abnormalities in T and B cell functions, increased apoptosis, tissue damage and multiorgan involvement, are satisfied (9). According to recent studies, there is growing evidence that immune factors can contribute to create a pro-inflammatory microenvironment that facilitates the persistence of endometriosis, giving the possibility to the endometrial implants to survive in ectopic sites (10, 11).

Indeed, the “classical” theory to explain the etiopathogenesis of endometriosis, are being, nowadays, questioned. In fact, some authors, are also suggesting that a potential role might be played by the so-called endocrine disrupting chemicals (EDCs), even though their role is still controversial. These EDCs, represented by heavy metals, pollutants, chemicals present in the air, water, and so on, seems to be able to interfere with many aspects of estrogen actions, including hormonal synthesis, modulation of receptors, agonist or antagonist action (12).

Some authors (13) have demonstrated significantly lower plasma levels of heavy metals, specifically lead (Pb), in patient with severe endometriosis, in confront of patients with mild or no endometriosis. For this reason, they have postulated a metabolically active role of lead in the endometriotic nodule. On the other hand, other authors (14), have postulated a relationship between IVF success and the degree of Pb contamination in the plasma and follicular fluid, with a demonstration of higher levels of intrafollicular Pb in the patients with endometriosis. Recently, another leading actor has been identified: the microbiome. The role of microbiota in the pathogenesis of endometriosis has been, in fact, object of several studies (15–17).

Considering the uterus as a non-sterile cavity, the refluxed menstrual effluent may carry bacteria, and contribute to inflammation, the establishment and growth of endometriotic lesions. With the word “microbiota” we referee to all the microorganisms coexisting in and on the body and it has a determining function in ensuring wellbeing (18). Changes in the normal composition, with an imbalance or impairment of the microbiota, can have severe consequences on health. Many pathologies have been found in deep correlation with alteration of microbiota, such as Polycystic Ovary Syndrome (PCOS), infertility, inflammatory bowel disease, psoriasis, arthritis, neuropsychiatric disease and even cancer (19–22); dysbiosis in the male reproductive tract microbiota can lead to seminal abnormalities, too (23, 24). And also, endometriosis seems to show complicate relation with altered microbiota. In this review we summarize the available literature data about the relationship between microbiota and endometriosis.

## Materials and methods

This narrative review was conducted following the search questions: “Does it really exist a relation between Dysbiosis and Endometriosis? How could it be explained?”. The search terms were extracted from published reviews and primary studies identified in a preliminary search. The search string was initially built by combining major keywords, i.e. Endometriosis, dysbiosis, microbiota, estrobolome, infertility. The search string was applied to the electronic literature databases MEDLINE (http://www.ncbi.nlm.nih.gov/pubmed) and WEB of SCIENCE (https://apps.webof-knowledge.com). Exclusion criteria accounted for (a) articles not written in English, (b) conference papers and reviews, (c) studies with information overlapping another publication (unless the overlapping study provided additional information useful for sensitivity analysis). In the event of overlapping studies, we selected the most recent and/or most comprehensive manuscript. The selection criteria of this narrative review included randomized clinical trials, non- randomized controlled studies (observational prospective, retrospective cohort studies, case-control studies, case series) and review articles on the possible effect of dysbiosis on endometriosis.

### Microbiota and endometriosis

The upper genital female reproductive tract has long been considered a sterile environment, even if a few years ago, some compartments such as the uterine cavity, has been discovered to have a local bacterial flora. Indeed, although no consensus has yet been achieved, it seems that some kind of resident microbiota is also present in these districts. The presence of bacterial DNA has been demonstrated in up to 95% of hysterectomy samples (25). Thus, while little is still known about the microbiota of the upper genital tract, the presence of a rich microbiota in the vagina is now well established. When analyzing vaginal microbiota, two main different situations can be found: a Lactobacillus-dominant environment (>90% Lactobacillus spp.) or a non-Lactobacillus-dominant environment (<90% Lactobacillus spp, >10% other bacteria) (26). The predominance of Lactobacillus spp. is associated with healthy vaginal microbiota, while imbalances lead to pathologies, such as bacterial vaginosis (27).

The role of Lactobacillus spp. is expressed through the production of lactic acid, that lowers the vaginal pH to ideal levels and inhibits different types of pathogenic microorganisms. Lactobacilli also produce hydrogen peroxide and bacteriocins that cooperate in their protective role (28).

When considering the lower and the upper genital microbiota, it looks that a non-Lactobacillus-dominance environment in the vagina may be associated with a worse reproductive outcome. Non-Lactobacillus-dominance environment may in fact trigger an inflammatory response in the endometrium, possibly explaining these results (26).

Of interest, dysbiosis, that consists in depletion of lactobacillus and overgrowth of pathogenic bacteria (Gardnerella, Prevotella, Bacteroides), may lead to damage to epithelial and mucosal barrier (29) and contribute to increase the risk for endometriosis, PID, endometritis and also infertility (15).

Several studies have suggested the correlation between endometriosis and dysbiosis, summarized in **Table 1**. In 2019, Ata B. et al. studied the composition of the gut, vaginal and cervical microbiota of stage III/IV endometriotic patients, comparing them with healthy controls and found a higher abundance of pathogenic species, including Gardnerella, Streptococcus, Escherichia, Shigella and Ureaplasma, in the cervical microbiota of endometriotic women (15).

**Table 1:** Main studies that have related endometriosis with microbioma alterations.

	Design	Population	Results
Ata B et al. (15)	Case-control	14 women with histology proven stage 3/4 endometriosis and 14 healthy controls	Increased pathogenic species (Gardnerella, Streptococcus, Escherichia, Shigella Ureaplasma) in patients with endometriosis
Khan KN et al. (16)	Case-control	36 patients with or without endometriosis	Abundance of Streptococcus, Moraxellae, Staphilococcus and Enterobacteria and lowered Lactobacillus in patients with endometriosis
Hernandes C et al. (17)	Case-control	10 patients with endometriosis and 11 healthy controls	High microbial diversity among ectopic endometrial lesions
Yuan M et al. (30)	Case-control on mice	N/A	Changes in gut microbiota of mice after developing endometriosis (increase in Firmicutes/Becteroidetes ratio)
Kyono K et al. (31)	Case-control	92 IVF patients (47 with Lactobacillus dominant microbiota, 45 with non-Lactobacillus dominant environment)	Higher pregnancy rate after restoring a vaginal Lactobacillus dominant environment
Khodaverdi S et al. (32) Itoh H et al. (33)	Case-placebo control Case-placebo control	37 patients, 18 randomized into LactoFem^®^ and 19 into placebo groups 66 patients, 29 randomized into L. gasseri OLL2809 and 32 into placebo group	Reduction of endometriosis-related pain after administration of Lactobacillus
Khan KN et al. (34)	Prospective observational	53 women with endometriosis and 47 control	Reduction of tissue inflammation, cell proliferation and angiogenesis after administration of Levofloxacine

Another study has demonstrated an elevated abundance of Streptococcus, Moraxellae, Staphilococcus and Enterobacteria species, together with a reduction of Lactobacillus, in women with endometriosis (26). Another aspect is that in patient with endometriosis, ectopic lesions seem to show higher microbial diversity (17). In a study on mice with endometriosis it was found that the gut microbiota changed after the development of endometriosis, with an increase in the Firmicutes/Bacteroidetes ratio (30).

Khan et al. in 2017 proposed the “bacterial contamination hypothesis” for pathogenesis of endometriosis, which involves the Lipopolysaccharide (LPS)/Toll-like receptor (TLR)4 cascade. They found high contamination of menstrual blood with E. coli in women with endometriosis, which cause constant source of bacterial endotoxin in the peritoneal fluid, linked to retrograde menstrual flow; this event could initiate the TLR4-mediated growth and progression of endometriosis and cause subsequent pelvic inflammation. LPS, as an inflammatory mediator, could be the initial trigger, and bacterial contamination could be its source in the intrauterine environment that could be the primary cause in the growth regulation of endometriosis (35). Although this theory paves the way for therapeutic strategies targeting bacterial endotoxin or TLR4 to suppress inflammation, their clinical application does not seem easily feasible.

In 2021, Jiang et al. hypothesizes that an alteration of the gut and reproductive tract microbiota can lead to an alteration of the immune response; indeed, dysbiosis disrupt the normal immune function, leading to the elevation of proinflammatory cytokines compromising the immunosurveillance and altering the immune cell profiles.In this way, this immune dysregulation can progress into a chronic state of inflammation, creating an environment that maintain the vicious cycle of endometriosis onset and progression (36).

Another interesting link between endometriosis and microbiota is the finding that bacterial contamination occurs in menstrual blood and endometrial samples together with increased occurrence of chronic endometritis (31, 37, 38). The possible occurrence of endometritis in women with endometriosis has not yet been clarified. However, microbiota modulation through antibiotics and probiotics could, in this perspective, be evaluated as an alternative target for endometriosis treatment.

In a recent study, some authors tried to restore a Lactobacillus-dominant environment using antibiotics and probiotics, reaching higher pregnancy rates (39). Also, treating endometritis with antibiotics seems to lead to improved reproductive outcomes (40). Antibiotics may indeed be a promising approach for treating endometriosis. This has already been demonstrated in animal models (32). Also, probiotic administration may lead to endometriosis improvement. Randomized, placebo-controlled trials have demonstrated the efficacy of oral administration of Lactobacillus in reducing endometriosis-associated pain in women (33). In mice, lactobacillus administration demonstrated improvement in regulation of immune system, increasing IL-12 e NK cell activity, reducing endometriotic lesions (34, 41, 42). A recent study has shown that the use of levofloxacin in patients with endometriosis is able to reduce tissue inflammation, cell proliferation and angiogenesis in endometrium and endometriotic lesions, also demonstrating improvement of ovarian endometrioma (34). These findings are relevant because reducing the risk of chronic endometritis may improve adverse reproductive outcome in women suffering from endometriosis. Unfortunately, the indiscriminate use of antibiotics is well known to carry several side risks, such as the selection of resistant species (43). Also, the resident microbiota should be well studied before targeting a specific antibiotic therapy (44).

### Estrobolome and endometriosis

The vaginal microbiota is influenced by a wide variety of factors, mainly hormonal, including contraceptive methods. Hormones in women play a critical role in changing the urogenital microbiome. For example, post-menopausal women losing estrogen activity can experience a decrease in the relative amounts of commensal species, resulting in several gynecological diseases (44). The name estrobolome refers to a collection of genes in the gut microbiome involved in estrogen metabolism (45). Estrobolome activity modulates the amount of excess estrogen circulating in the body (46). When this activity is impaired, it is generally due to an imbalance in the gut microbiome, therefore the excess of estrogen can be retained in the body and enrich endometrial and peritoneal environment (47).

Alteration in microbial communities in endometriosis could both trigger development and maintenance of the lesions. For this reason, it is worth wondering whether hormonal contraceptive pills, commonly used for the treatment and prevention of endometriosis, might have a role in regulating dysbiosis. Few studies have been conducted on this subject.

A study of 2018 conducted on 101 women, found a statistically significant association between combined oral contraceptive pill and vaginal microbiota. It found that combined hormone therapy restores a normal vaginal status and corrects the alteration (48).

A successive study conducted in 2022 on 208 women confirmed these findings. Hormone administration corrected the dysbiosis and preserved a normal vaginal status in the patients involved (49).

These findings may be an interesting field of study also in patients with endometriosis. In this subpopulation, interesting data emerged from the study by Le at al. in 2021 (50). The authors investigated estrogen metabolites as well as microbial phenotypes in patients with endometriosis and in healthy control. They then examined the effects of surgery with or without hormonal therapy on estrogen and microbial profiles. They found that patients with endometriosis showed a peculiar dysbiosis both in gastrointestinal tract and genitourinary tract, together with an alteration in estrogen metabolism. They also found out that oral contraceptive pill administration was related to microbial changing, even though these finding needs further studies.

In this way it seems more understandable how dysbiosis, defined as imbalance or impairment of the microbiome, may be involved in disease progression.

Previous studies have reported alterations of the bacterial microbiome in the reproductive tract even of adenomyosis women, and a recent study focused on the direct comparison of vaginal microbiota between women with and without adenomyosis. It was found out that a direct relationship between genital tract microbiome and adenomyosis exists: an increase in microbial richness seems to correlate with adenomyosis, maybe inducing a change of certain vaginal microbiota which can introduce chronic inflammation that leads to adenomyosis (51–54).

### The role of the microbiome in the pathogenesis of pain

As already stated, the gastrointestinal tract communicates bidirectionally with the central nervous system through the so called gut-brain-axis (GBA) (55, 56). The microbiota participates in supplying the gut with nutrients and maintaining its barrier integrity. The main neurotransmitter that regulates this communication is serotonin (57).

Alteration in gut microbiome has been related to gastrointestinal symptoms and disorders, such as inflammatory bowel disease (IBD) or irritable bowel syndrome (IBS) and even cancer (58). It seems that short-chain fatty acid (SCFAs) may have, on the contrary, a protective role inducing and promoting colonization of the gut by protective bacteria (59). SCFAs are microbial metabolites that plays a role in the inflammation control acting of the T-regulatory cells (60).

Nociceptive pain is modulated by multiple neurons both centrally and peripherally by substances whose production is affected by microbiome. In particular, neuropathic pain, which often occurs as a result of nerve-damaging trauma and progressively leads to central sensitization of chronic pain, seems to be perpetrated by a neuroimmune activation that might be influenced by microbiome (61). The gut microbiota in particular regulates microglial function, but it can affect plenty of other cells, such as astrocytes, endothelial cells, monocytes, and T-cells (62).

When these cells are activated, they produce multiple pro-inflammatory mediators, such as interleukin-1 *β*, interferon-*γ*, TNF- *α*, that influence synaptic neurotransmission, increasing glutamate and reducing GABA (63). Also in endometriosis, as discussed previously, the gastrointestinal tract disrupts immune function, leading to elevation of inflammatory cytokines ad immune system alteration. Recent discoveries regarding the gut microbiome and visceral pain led to hypotheses about the correlation between chronic pelvic pain (CPP), that affects women with endometriosis, and human microbiota. In a study conducted by Shoskes et al, it was determined that patients with CPP had lower gut microbiota diversity than the control group (64). Pelvic allodynia may also be influenced by lowered levels of acyloxyacyl hydrolase (AOAH), expressed by microglia. In one study it was suggested that AOAH plays a role in the modulation of pelvic pain and it is strictly related to gut microbiome, which influences its production (65). Moreover, one of the SCFAs produced by microbiome, butyrate, has been proposed as an agent with indirect effect on regulating visceral inflammatory pain. Probiotics are living microorganisms that can provide benefits to the host, and some of the most recent studies suggest that probiotics may improve chronic intestinal disorders, and even pelvic visceral pain (66). However, further evaluation of their efficacy is still needed.

## Conclusion

The relationship between endometriosis and the human microbiota is still enigmatic. Many studies in the literature has conducted poor quality data, with consistent confounding factors. However, there still seems to be a close correlation. Endometriosis is a widely spread disease that has a serious impact on quality of life. Finding new strategies to treat this disease is of utmost importance. In particular, future strategies should aim to convert a state of dysbiosis into a favorable genital microenvironment; to do so, the possible use of antibiotics, probiotics, prebiotics, up to and including fecal, vaginal, or uterine microbial transplantation has been theorized, all so far with controversial results. Therefore, understanding the role of the microbiota could be a key to better understanding disease pathogenesis and progression. Future perspectives are needed to explain the mechanisms underlying this association.

## Author contributions

VF, AG, and SM performed the literature re-search. BZ and VF wrote the manuscript, BZ and AS made the final revision. All authors contributed to editorial changes in the manuscript. All authors contributed to the article and approved the submitted version.
